# Drug resistance of oral bacteria to new antibacterial dental monomer dimethylaminohexadecyl methacrylate

**DOI:** 10.1038/s41598-018-23831-3

**Published:** 2018-04-03

**Authors:** Suping Wang, Haohao Wang, Biao Ren, Xiaodong Li, Lin Wang, Han Zhou, Michael D. Weir, Xuedong Zhou, Radi M. Masri, Thomas W. Oates, Lei Cheng, Hockin H. K. Xu

**Affiliations:** 1 State Key Laboratory of Oral Diseases & National Clinical Research Center for Oral Diseases & Deptartment of Cariology and Endodonics West China Hospital of Stomatology, Sichuan University, Chengdu, 610041 China; 2 Department of Advanced Oral Sciences and Therapeutics, University of Maryland Dental School, Baltimore, MD 21201 USA; 3 Department of Oral Medicine, School of Stomatology, Zhejiang University, Hangzhou, Zhejiang, China; 4 VIP Integrated Department, Stomatological Hospital of Jilin University, Changchun, China; 5 Maurice H. Kornberg School of Dentistry, Temple University, Philadelphia, PA 19140 USA; 6 Center for Stem Cell Biology & Regenerative Medicine, University of Maryland School of Medicine, Baltimore, MD 21201 USA; 7 Department of Mechanical Engineering, University of Maryland Baltimore County, Baltimore County, MD 21250 USA; 8 Member, Marlene and Stewart Greenebaum Cancer Center, University of Maryland School of Medicine, Baltimore, MD 21201 USA

## Abstract

Only two reports exist on drug-resistance of quaternary ammonium monomers against oral bacteria; both studies tested planktonic bacteria for 10 passages, and neither study tested biofilms or resins. The objectives of this study were to investigate the drug-resistance of *Streptococcus mutans*, *Streptococcus sanguinis* and *Streptococcus gordonii* against dimethylaminohexadecyl methacrylate (DMAHDM), and to evaluate biofilms on resins with repeated exposures for 20 passages for the first time. DMAHDM, dimethylaminododecyl methacrylate (DMADDM) and chlorhexidine (CHX) were tested with planktonic bacteria. Biofilms were grown on a resin containing 3% DMAHDM. Minimum-inhibitory concentrations were measured. To detect drug-resistance, the survived bacteria from the previous passage were used as inoculum for the next passage for repeated exposures. *S. gordonii* developed drug-resistance against DMADDM and CHX, but not against DMAHDM. Biofilm colony-forming units (CFU) on DMAHDM-resin was reduced by 3–4 log; there was no difference from passages 1 to 20 (p > 0.1). No drug-resistance to DMAHDM was detected for all three bacterial species. In conclusion, this study showed that DMAHDM induced no drug-resistance, and DMAHDM-resin reduced biofilm CFU by 3–4 log, with no significant change from 1 to 20 passages. DMAHDM with potent antibacterial activities and no drug-resistance is promising for dental applications.

## Introduction

Dental caries is a prevalent biofilm-infectious disease and a major public health problem globally with heavy economic burdens^1^. The shift in microbial balance of dental plaque biofilms could lead to increased proportions of acid-producing and acid-tolerant bacteria, *e.g*. streptococci strains. This can reduce the plaque pH, resulting in tooth demineralization and caries^2^. Restorative materials are used to fill tooth cavities^3^. However, complete removal of caries tissues is difficult, and there are usually residual bacteria in the affected tooth tissues^4^. The residual and newly-attached bacteria can degrade the polymeric components of the resins and compromise the marginal integrity. This can lead to recurrence of caries (secondary caries), which has been reported as a main reason for restoration failures^5^. Therefore, restorative materials with antimicrobial functions are advantageous in order to prevent the spread of caries after completing the restoration, to inhibit recurrent decay and to reduce the restoration failure rates^6^.

Antibacterial agents such as chlorhexidine (CHX)^7^ and silver nanoparticles^8^ were incorporated into dental restorative materials including composites, glass ionomer cements and adhesives^9–13^. Furthermore, quaternary ammonium monomers (QAMs) were incorporated into dental resins^14–20^. QAMs could be covalently bonded and immobilized in resins to provide long-term antimicrobial functions, without being leached out and lost over time such as CHX^16^. Previous studies developed novel QAMs including 12-methacryloyloxydodecylpyridinum bromide (MDPB)^17^, quaternary ammonium polyethylenimine (QPEI)^18^, quaternary ammonium dimethacrylate (QADM)^19^, and dimethylaminododecyl methacrylate (DMADDM)^20^. Recently, dimethylaminohexadecyl methacrylate (DMAHDM) were synthesized and incorporated into resins, showing potent antibacterial effects, long-term effectiveness, and flora modulation capacity^21,22^. QAMs possess a broad spectrum of antimicrobial effect against a wide range of microorganisms^23^. The antibacterial mechanism of quaternary ammonium compounds is related to the interaction between their cationic properties and the cell membranes. When the negatively-charged bacteria cells contact the positive quaternary amine charge (N^+^), the disturbed electric balance causes the disruption of bacterial cell wall and leakage of the cytoplasm^23^. Several QAMs were incorporated into dental materials, showing effectiveness in suppressing the growth and metabolism of oral biofilms^24–27^.

Although antibacterial dental materials containing QAMs have promising clinical benefits, little attention has been paid to the emergence of potential antibacterial drug resistance in oral microorganism induced by QAMs. To date, there has been no report on any *in vivo* study on oral bacterial resistance to QAMs^28^. A literature search revealed only two *in vitro* reports on the drug resistance of QAMs against oral microbes^29,30^. One report showed that *Enterococcus faecalis* (*E. faecalis*) and *Streptococcus mutans* (*S. mutans*) did not develop resistance to MDPB with ten repeated exposures^29^. The other study investigated eight common oral bacteria species; no species developed resistance to DMAHDM, and only *Streptococcus gordonii* (*S. gordonii*) developed a mild resistance to DMADDM with 10 passages^30^. These two studies only investigated planktonic bacteria, not biofilms. It has been reported that bacteria embedded in a biofilm are up to 1000-fold more resistant to antibiotics compared to their planktonic counterparts^31^. In addition, in these two studies^29,30^, the QAM was dissolved in the culture medium, not co-polymerized in a dental resin as a clinically-relevant restorative material. To date, a literature search revealed no report on: (1) investigation of drug resistance in oral or non-oral biofilms induced by QAMs (previous studies only tested planktonic bacteria); (2) testing of bacterial drug resistance for QAM-containing resins (previous studies only added QAM into culture medium); and (3) evaluation of relatively longer-term drug resistance with repeated exposures to a QAM for more than 10 passages.

Therefore, the objectives of this study were to investigate the drug resistance of oral bacteria against QAM in both planktonic and biofilm forms, with DMAHDM being co-polymerized in a dental resin and having repeated exposures for 20 passages. While DMADDM and CHX were included as comparative controls, DMAHDM was the focus of the present study because DMAHDM was strongly antibacterial, had excellent biocompatibility, and did not compromise the mechanical and physical properties of the resins^20,32,33^. It was hypothesized that: (1) Different antibacterial agents (DMAHDM, DMADDM, CHX) would induce different levels of drug resistance in planktonic bacteria; (2) Different oral bacterial species would exhibit different drug resistance properties against the same antibacterial agent; (3) The new DMAHDM would not induce drug resistance in planktonic bacteria and biofilms, whether being incorporated into the culture medium or co-polymerized in a dental resin, from passages 1 to 20.

## Material and Methods

### Synthesis of DMAHDM and DMADDM

DMAHDM was synthesized using a modified Menschutkin reaction^33^. Briefly, 10 mmol of 2-(dimethylamino) ethyl methacrylate (DMAEMA, Sigma-Aldrich, MO, USA) and 10 mmol of 1-bromohexadecane (BHD, TCI America, Portland, OR, USA) were combined with 3 g of ethanol in a 20 mL scintillation vial. The vial was stirred at 70 °C for 24 h for the reaction to occur. The solvent was then evaporated, yielding DMAHDM as a clear, colorless, and viscous liquid^33^. Similarly, 10 mmol 2-bromoethyl methacrylate (BEMA, Sigma-Aldrich) and 10 mmol 1-(dimethylamino) dodecane (DMAD, Sigma-Aldrich) were added in a 20 mL vial which was capped and stirred at 70 °C for 24 h. After the reaction was completed, the solvent was evaporated to yield DMADDM as a clear and viscous liquid^34^.

### Bacterial strains and culture

All bacterial experiments were approved by the University of Maryland Baltimore Institutional Review Board. Three oral streptococcal species were obtained from the American Type Culture Collection (ATCC, Manassas, VA, USA): *S. mutans* UA159; *Streptococcus sanguinis* (*S. sanguinis*) ATCC 10556, and *S. gordonii* DL1. They were cultured in brain-heart infusion broth (BHI, Difco, Sparks, MD, USA) at 37 °C with 5% CO_2_. *S. gordonii* and *S. sanguinis* were selected because they are pioneer colonizers of oral biofilms. *S. mutans* was selected as it is the main etiologic agent causing tooth caries.

### Minimal inhibitory concentration (MIC) measurements

Serial two-fold microdilution method was used to determine the MIC following a previous method^30^. The MIC is defined as the lowest concentration of an antibiotic that prevents the visible growth of planktonic bacterial cells. DMAHDM or DMADDM was dissolved in sterile distilled water at 200 μg/mL. CHX served as the control with a starting concentration of 250 μg/mL. Serial two-fold dilutions of each solution were added into 96-well plate with 100 μL in each well. Overnight bacterial suspension was adjusted to 10^6^ CFU/mL, and 100 μL was inoculated into each well of the 96-well plate. After incubation at 37 °C with 5% CO_2_ for 2 days, MIC was determined: The lowest concentration at which no visible bacterial growth appeared was regarded as the MIC. All tests were repeated in three replicates.

### Planktonic bacterial drug resistance assay

To investigate the drug resistance of planktonic bacteria induced by the antibacterial agents, 20 passages of sub-MIC measurements were performed. Using DMAHDM as an example, this sub-MIC value was 1/2 of the MIC of DMAHDM. 100 μL of bacterial suspension in the sub-MIC well was taken and inoculated into 10 mL of fresh medium at 37 °C with 5% CO_2_ overnight. Then the overnight bacterial suspension was diluted to a concentration of approximately 10^6^ CFU/mL for the next MIC test. This constituted passage 1 for the bacteria to be exposed to DMAHDM^30^. Then, 100 μL of the DMAHDM-exposed bacterial suspension in the sub-MIC well was taken, inoculated in 10 mL of fresh medium at 37 °C with 5% CO_2_ and cultured overnight to expand the bacteria, without DMAHDM. In this way, the bacteria that survived the DMAHDM exposure during passage 1 were expanded overnight (without exposure to DMAHDM) to approximately 10^9^ to 10^10^ CFU/mL. This surviving bacterial suspension was then diluted to approximately 10^6^ CFU/mL and used for the next MIC measurement with exposure to DMAHDM, which constituted passage 2. The MIC tests were repeatedly performed for 20 such passages. It took about two months to complete 20 passages. The reason to perform 20 passages, each using the surviving bacteria with exposure to DMAHDM, was to investigate the drug resistance with a relatively long-term exposure to the antibacterial agent. With such repeated exposures to the antibacterial agent, any increase in MIC with increasing passage number would indicate bacterial drug resistance^35^. DMADDM and CHX were tested in the same manner. Each bacterial species was tested respectively and all three species were tested in the same manner. The tests were repeated in three replicates.

### Preparation of DMAHDM resin disks

To test biofilms growth on the polymerized resin surfaces, resin disks containing DMAHDM were fabricated. DMADDM and CHX were not used in resin disks because the results from the previous section indicated that both of them induced drug resistance and therefore would not be preferred for dental resin applications. In addition, CHX cannot be co-polymerized in the resin and will leach out and be lost over time in the culture medium. DMAHDM did not induce any drug resistance according to the previous section, and can be polymerized in resins with potent antibacterial functions^36–38^, and hence would be promising for clinical applications. Therefore, DMAHDM was selected for the following drug resistance experiments of biofilms on resins.

The resin consisted of bisphenol A glycidyl dimethacrylate (BisGMA, Esstech, Essington, PA) and triethylene glycol dimethacrylate (TEGDMA, Esstech) at a mass ratio of 1:1. The mixture was rendered light-curable with 0.2% camphorquinone and 0.8% ethyl 4-N,N-dimethylaminobenzoat^36^. Following previous studies^36^, DMAHDM was mixed into resin at a DMAHDM/(resin + DMAHDM) mass fraction of 3%. Following a previous study^37^, the cover of a sterile 48-well plate (Costar, Corning Inc., Corning, NY, USA) was used as molds to make resin disks with approximately 10 mm in diameter and 1 mm in thickness, which were photo-cured for 1 minute. The cured resin disks were immersed in distilled water at 37 °C with stirring for 24 h to remove the initial burst release of unpolymerized monomers, and then sterilized by ethylene oxide before use. Control disks of the same resin with 0% DMAHDM were fabricated and treated in the same manner.

### Serial anti-biofilm assay

The previous section tested planktonic bacterial drug resistance with the antibacterial agent dissolved in the culture medium. In this section, mature biofilms were grown on resin surfaces containing the antibacterial agent co-polymerized in the resin. Briefly, *S. mutans*. *S. sanguinis* or *S. gordonii* were cultured overnight in BHI medium at 37 °C with 5% CO_2_. The overnight-cultured bacterial suspensions were diluted by BHI plus 1% sucrose medium to obtain the inoculum for biofilm formation, as previously reported^39,40^. For passage one, the overnight bacterial suspension was diluted to a concentration of 10^6^ CFU/mL. Then 1.5 mL of the suspension was inoculated into each well of 24-well plates. Each well contained a resin disk on the bottom. After 24 h, the disks with adherent biofilms were transferred to new 24-well plates with fresh BHI medium, and incubated for another 24 h. This totaled 48 h of incubation, which was previously shown to form relative mature biofilms on resins^36–38^. Most of the bacteria in the biofilms on DMAHDM-containing resin disks were killed, while some bacteria survived^36–38^. This 2-day biofilm on the resin disk with DMAHDM was washed and collected; this is referred to as the “DMAHDM resin-challenged-and-survived bacteria”. This constituted passage 1. This survived bacterial suspension was divided into two parts. Part one was resuspended into 1 mL PBS solution and then serially diluted for CFU determinations. Part two (100 μL) of the DMAHDM resin-challenged-and-survived bacteria suspension was inoculated into 10 mL fresh BHI medium and cultured overnight to expand the bacteria to be used for passage 2. This overnight culture (without exposure to DMAHDM) yielded 10^9^ to 10^10^ CFU/mL. This suspension was diluted and re-inoculated into 24-well plates with resin disks with 10^6^ CFU/mL, and cultured for 2 days as described above, which constituted passage 2. Thereafter, for each subsequent passage, the survived bacteria in 2-day biofilms on the DMAHDM resin from the previous passage were collected, expanded overnight, and re-inoculated onto new resin disks. This procedure was repeated for 20 passages, which took nearly 4 months to finish, to evaluate the relatively long-term drug resistance on the DMAHDM resin. Resin disks with 0% DMAHDM were tested in the same manner as control. Each bacterial species was tested separately and the three species were tested in the same manner.

For the CFU measurement, the 2-day biofilm on each resin disk was harvested by scraping with a sterilized surgical scalpel blade (3 M, St Paul, MN, USA) into 1 mL phosphate buffered saline (PBS) buffer, which was vortexed for 1 min, followed by water bath sonication for 5 min. Then the biofilm suspensions were serially 10-fold diluted, spread onto BHI agar plate and incubated at 37 °C with 5% CO_2_ for 2 days^38^. Then the number of colonies was measured in the same manner as in previous studies^36–38^. The CFU reduction = CFU of biofilm on control resin with 0% DMAHDM – CFU of biofilm on resin with 3% DMAHDM.

### Statistical analysis

All statistical analyses were performed using statistical software SPSS 21.0 (SPSS, Chicago, IL, USA). MIC values and changes in the reduction of CFU/mL for every bacterial strain at every passage were compared with each other using one-way analyses of variance (ANOVA). Paired t-testing was performed to compare the CFU/mL difference between experiment and control group at every passage. Differences were considered statistically significant at the 5% level (p < 0.05).

## Results

### Resistance assay of antibacterial agents against *S. mutans*

**Figure 1:**
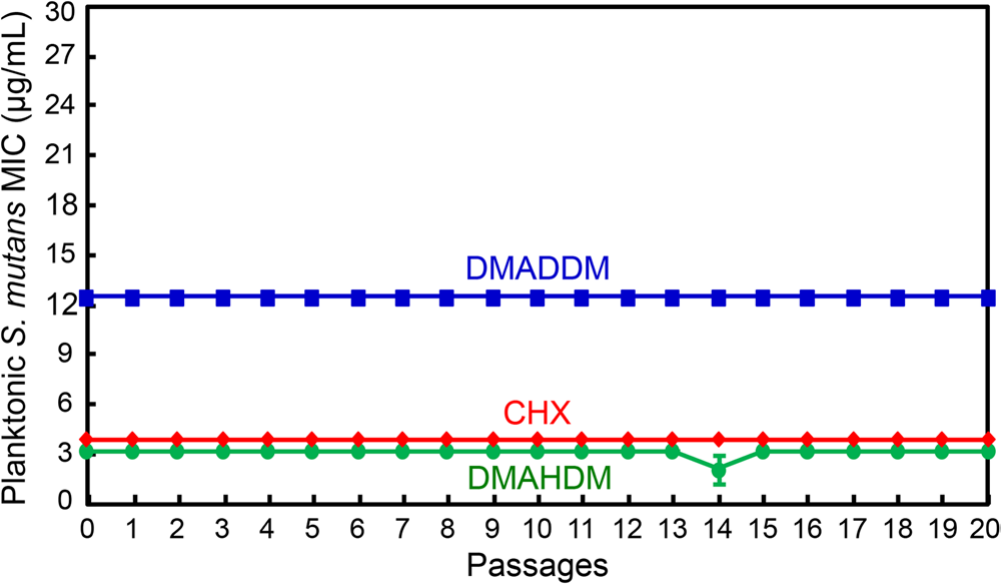
Minimal inhibitory concentration (MIC) of DMAHDM, DMADDM and CHX dissolved in culture medium against planktonic *S. mutans* (mean ± SD; n = 3). MIC is the lowest concentration of an antimicrobial agent at which bacterial growth is completely inhibited. MIC was measured from passages 0 to 20. Each subsequent passage used the surviving bacteria from the previous passage that were exposed to the antibacterial agent, in order to test drug resistance. DMAHDM, DMADDM and CHX induced no drug resistance in *S. mutans* in 20 passages.

The serial MICs of planktonic *S. mutans* after exposure to DMAHDM, DMADDM and CHX were measured for 20 passages (Fig. 1). The MIC was 3.125 µg/mL for DMAHDM, 12.5 µg/mL for DMADDM, and 3.9 µg/mL for CHX. They remained the same from passage 1 to 20. The unchanged MIC values indicate that *S. mutans* did not develop resistance to DMAHDM, DMADDM or CHX.

### Resistance assay of antibacterial agents against *S. sanguinis*

**Figure 2:**
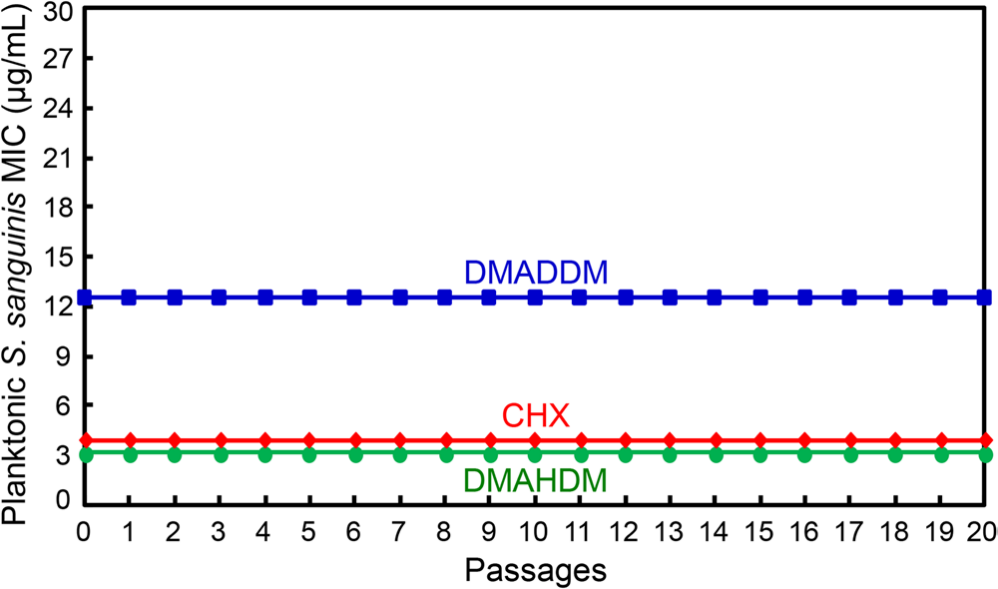
Minimal inhibitory concentration (MIC) of DMAHDM, DMADDM and CHX dissolved in culture medium against planktonic *S. sanguinis* (mean ± SD; n = 3). During the 20 passages, each subsequent passage used the surviving bacteria from the previous passage as inoculum that were exposed to the antibacterial agent, in order to test drug resistance. *S. sanguinis* developed no drug resistance against DMAHDM, DMADDM and CHX.

Similarly, the drug resistance results for planktonic *S. sanguinis* are plotted in Fig. 2. There was no increase in MIC in 20 passages of exposure to DMAHDM, DMADDM and CHX, with stable MIC at 3.125 µg/mL for DMAHDM, 12.5 µg/mL for DMADDM, and 3.9 µg/mL for CHX. These results demonstrated that these three antibacterial agents did not induced drug resistance in *S. sanguinis*.

### Resistance assay of antibacterial agents against *S. gordonii*

**Figure 3:**
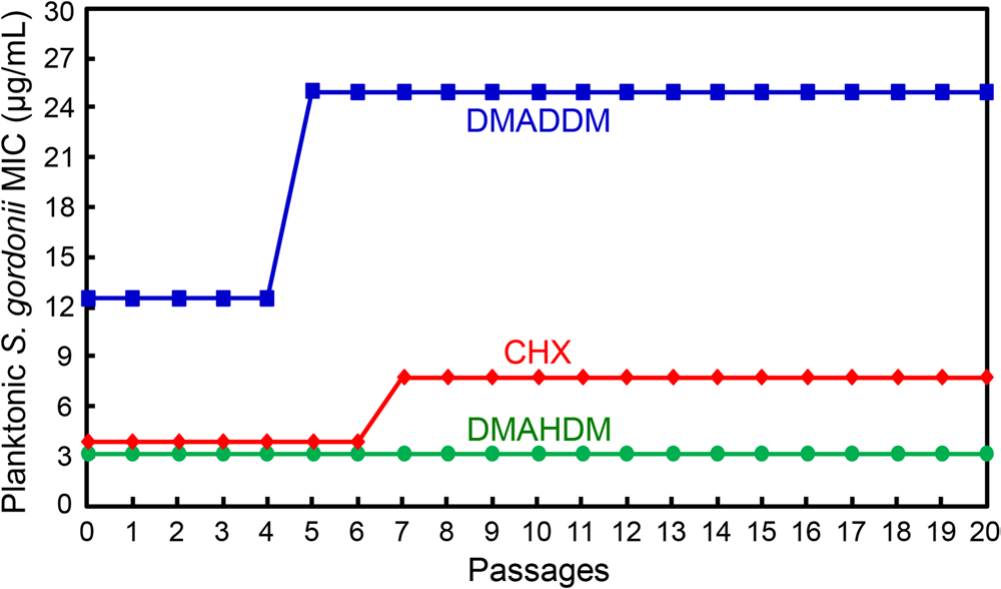
Minimal inhibitory concentration (MIC) of DMAHDM, DMADDM and CHX dissolved in medium against planktonic *S. gordonii* (mean ± SD; n = 3). Twenty passages were tested, and each subsequent passage used the surviving bacteria from the previous passage as inoculum, in order to examine potential drug resistance. A two-fold increase in MIC was found for *S. gordonii* against DMADDM or CHX, indicating drug resistance. However, no changes in MIC occurred for DMAHDM, indicating no drug resistance.

The results changed for *S. gordonii* (Fig. 3). For DMADDM, the MIC of planktonic *S. gordonii* increased from 12.5 µg/mL to 25 µg/mL at passage 5, and remained at 25 µg/mL till passage 20. For CHX, the MIC increased from 3.9 µg/mL to 7.8 µg/mL at passage 7, and then stayed constant till passage 20. In contrast, the MIC stayed constant at 3.125 µg/mL for DMAHDM. These results demonstrate that DMADDM and CHX induced bacterial resistance in *S. gordonii*; however, DMAHDM induced no drug resistance.

### Serial anti-biofilm assay of *S. mutans*

**Figure 4:**
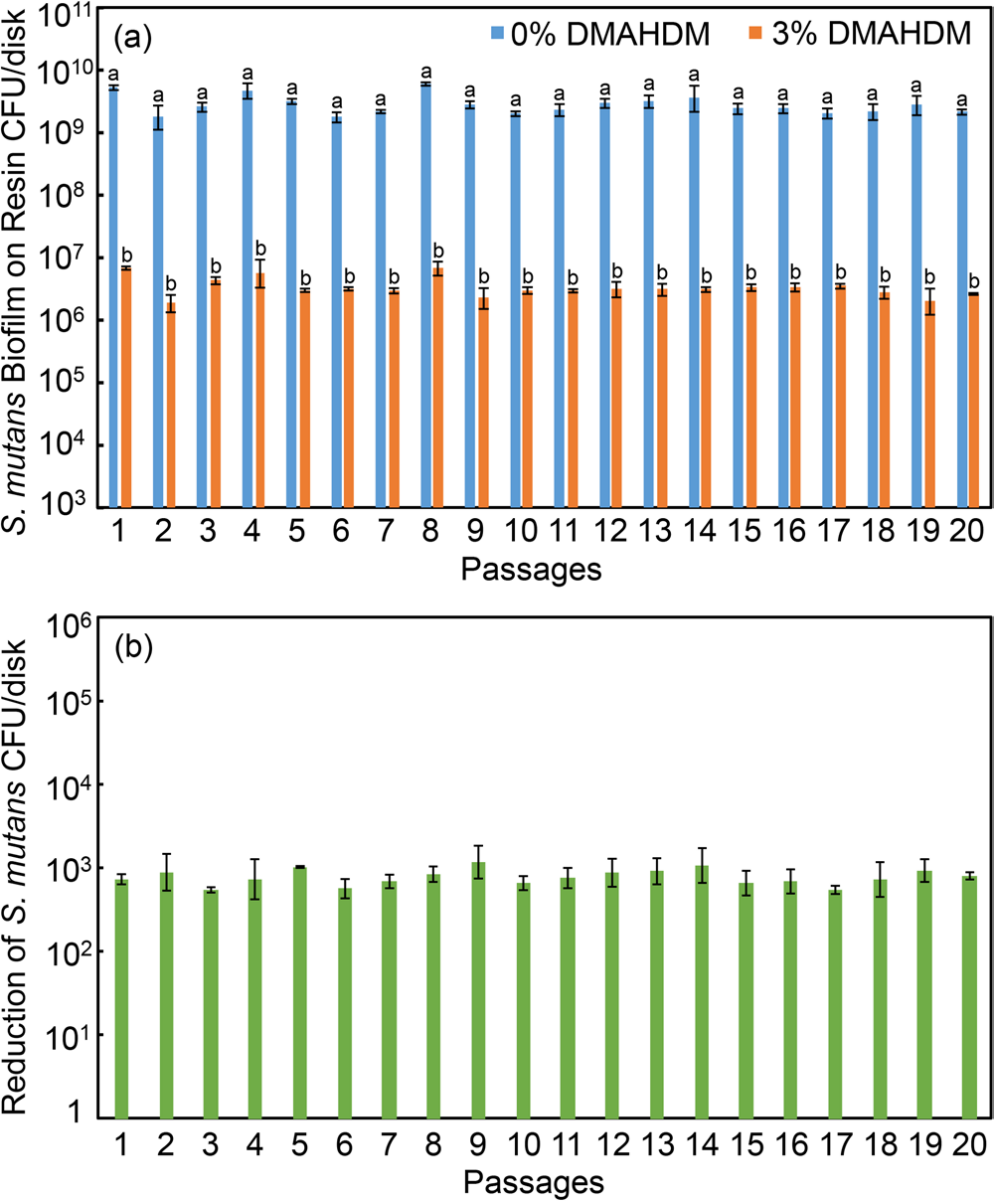
Colony-forming units (CFU) of *S. mutans* biofilms on resins with 0% or 3% DMAHDM (mean ± sd; n = 6). To evaluate drug resistance to DMAHDM, 20 passages were tested, each using the surviving bacteria from the previous passage as inoculum. From 1 to 20 passages, the CFU on the DMAHDM resin was maintained to be 3 log less than that without DMAHDM, indicating no drug resistance. In (**a**), bars with dissimilar letters are significantly different from each other (p < 0.05). In (**b**), all values are similar (p > 0.1).

To examine whether the DMAHDM-containing resin would induce drug resistance for *S. mutans* biofilms formed on the resin surface, serial anti-biofilm effects of DMAHDM resin were measured for 20 passages (Fig. 4). In (A), the biofilm CFU on the DMAHDM resin were substantially reduced, compared to the control resin without DMAHDM. The CFU counts of *S. mutans* biofilms were reduced by about 3 log via the DMAHDM resin. In (B), regarding the net reduction in biofilm CFU counts, there was no significant difference in the extent of CFU reduction from passage 1 to 20 (p > 0.1). These results demonstrate that no drug resistance was induced in *S. mutans* biofilms after repeated exposure in 20 passages to the DMAHDM resin.

### Serial anti-biofilm assay of *S. sanguinis*

**Figure 5:**
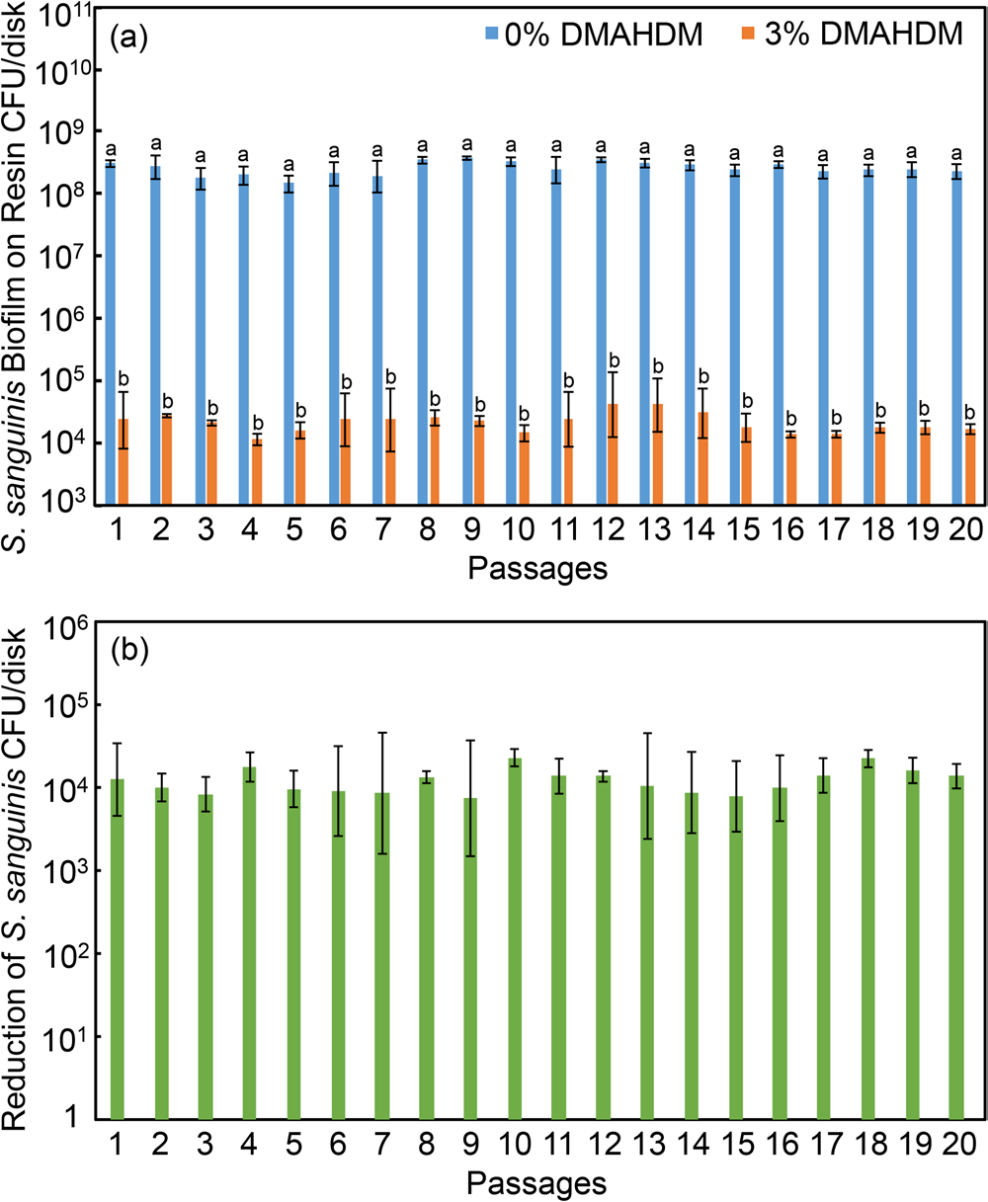
*S. sanguinis* CFU in biofilms on resins containing 0% or 3% DMAHDM (mean ± sd; n = 6). To evaluate drug resistance to DMAHDM, 20 passages were tested, each using the surviving bacteria from the previous passage as inoculum. From 1 to 20 passages, CFU on DMAHDM resin was 4 log less than that without DMAHDM, indicating no drug resistance. In (**a**), bars with dissimilar letters are significantly different from each other (p < 0.05). In (**b**), all values are similar (p > 0.1).

The *S. sanguinis* biofilm results on resins are plotted in Fig. 5: (A) CFU counts of *S. sanguinis* biofilms grown on resins with 0% or 3% DMAHDM, and (B) the extent of reduction in biofilm CFU via the DMAHDM resin. The majority of *S. sanguinis* biofilm population was killed, with the reduction in CFU remaining stable, at about 4 log. Approximately 99.99% of the bacteria in the three-dimensional biofilm on the DMAHDM resin were compromised. After each passage, the remaining 0.01% bacterial suspension was expanded and then re-challenged by the DMAHDM resin for the next passage, yielding a similarly substantial reduction in CFU. These data show that the DMAHDM resin induced no drug resistance in *S. sanguinis* biofilms.

### Serial anti-biofilm assay of *S. gordonii*

**Figure 6:**
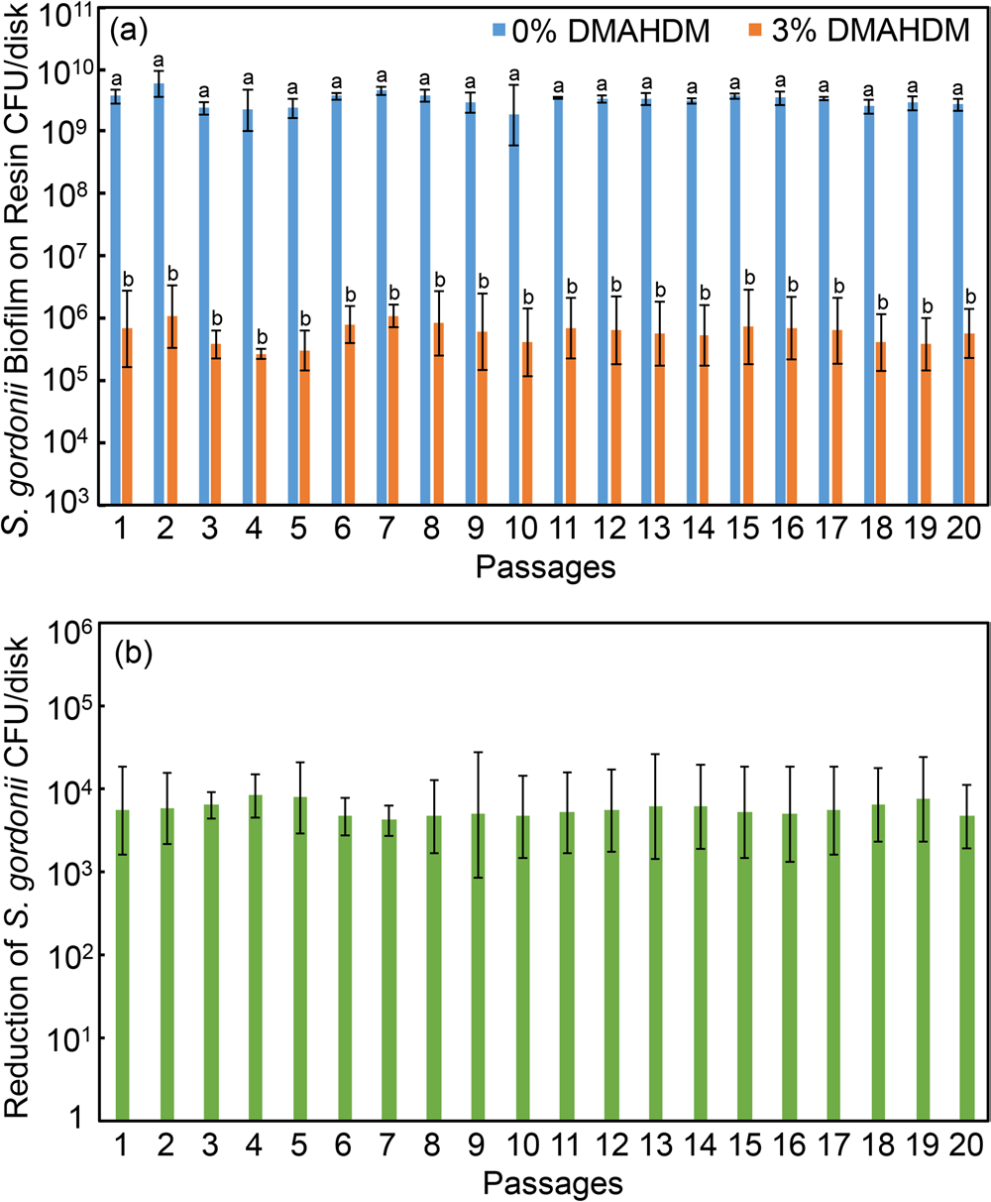
*S. gordonii* biofilm colony-forming units (CFU) on resin with 0% or 3% DMAHDM (mean ± sd; n = 6). To evaluate drug resistance to DMAHDM, 20 passages were tested, each using the surviving bacteria from the previous passage as inoculum. From 1 to 20 passages, CFU on DMAHDM resin was maintained at 4 log less than that without DMAHDM, demonstrating no drug resistance. In (**a**), bars with dissimilar letters are significantly different from each other (p < 0.05). In (**b**), all values are similar (p > 0.1).

For *S. gordonii*, the results are shown in Fig. 6 with 2-day biofilms on resin disks containing 0% or 3% DMAHDM: (A) biofilm CFU, and (B) biofilm CFU reduction via DMAHDM resin. Figure 6a showed that in each passage, adding DMAHDM into the resin substantially decreased the biofilm biomass, with a nearly 4 log reduction in CFU. Hence, almost 99.99% of the bacteria in the biofilms were compromised by the DMAHDM resin, compared to that with 0% DMAHDM. Figure 6b showed that there was no obvious change in the extent of CFU reduction after repeatedly passaging the survived bacteria with repeated exposure to the DMAHDM resin. These results demonstrate that the *S. gordonii* biofilms remained highly and similarly susceptible to the DMAHDM resin from passage 1 to 20.

## Discussion

This study investigated the antibacterial drug resistance of DMAHDM against three oral bacterial strains (*S. mutans*, *S. sanguinis* and *S. gordonii*) in planktonic and biofilm forms using repeated exposures for 20 passages. The results suggest that all hypotheses were proven: Different antibacterial agents (DMAHDM, DMADDM, CHX) caused different levels of drug resistance in planktonic bacteria; different species had different drug resistance properties against the same antibacterial agent; and DMAHDM induced no drug resistance from passages 1 to 20. It should be noted that we performed our experiments with triplicates at the same time, without repeating them on different days. In addition, a 2-fold increase in MIC indicates a moderate drug resistance, not a substantial drug resistance. Therefore, further studies are still needed to investigate the oral bacterial drug resistance against various antibacterial dental materials. Nonetheless, the different oral bacteria species demonstrated different drug resistance properties, with *S. gordonii* acquiring resistance to DMADDM and CHX, but *S. mutans* and *S. sanguinis* showing no resistance, after the same repeated challenges. In addition, DMAHDM caused no drug resistance in planktonic bacteria and their biofilms, whether dissolved in culture medium or co-polymerized in a dental resin, for all three species tested. The substantial 3–4 log reduction in biofilm CFU growing on DMAHDM resin disks remained the same from passage 1 to 20, each subsequent passage using the survived bacteria from the previous passage as inoculum with repeated exposures to DMAHDM. Therefore, the resin containing 3% DMAHDM had highly potent antibacterial efficacy against biofilms of all three species, and the biofilms remained highly and similarly sensitive to the DMAHDM resin after repeated exposures for 20 passages. These results indicate that the resin containing 3% DMAHDM is promising for dental applications.

Antibacterial resistance has been a significant global concern; it increases the cost of health care and threatens the effectiveness in the treatment of common infectious diseases caused by bacteria, fungi, *et al*.^41^. Bacteria that are resistant to antibiotics could grow in the presence of higher drug concentrations. Hence, resistant bacteria require greater MIC values than the ones which are sensitive to the antibiotic. The present study showed that there was no drug resistance among planktonic *S. mutans* and *S. sanguinis* against DMAHDM, DMAHDM or CHX during repeated exposures to sub-inhibitory concentrations. *S. gordonii* developed a moderate drug resistance to DMADDM and CHX, but no drug resistance to DMAHDM. Indeed, *S. gordonii* was reported to show resistance to penicillin (β-lactam antibiotics, act by inhibiting penicillin-binding proteins and preventing new cell wall formation), with the MIC increasing by more than 100 folds, which was thought to involve multiple mutations in both penicillin-binding proteins (PBPs) and non-PBP genes^42^. Antibiotic resistance is a natural phenomenon and some drug resistance occurs without human action. However, the overuse and abuse of antibiotics could lead to the emergence of higher-level antibiotic-resistant bacteria. Therefore, the drug resistance induction in the present study was not enough to produce high-level DMADDM/CHX resistant *S. gordonii*. The impermeability of *S. gordonii* cell membrane to DMADDM may contribute to the potential resistance mechanism, as showed in a previous study^30^. Furthermore, the possible different target site mutations of the DMADDM-resistant and CHX-resistant *S. gordonii* may also help explain the relatively low level of drug resistance. Further studies such as whole genome sequencing are needed to detect the possible mutation sites or genes in the resistant bacteria.

The results of the present study indicate that CHX induced a moderate drug resistance; this result was consistent with a previous study showing that CHX induced drug resistance^43^. That study investigated strains associated with periodontitis, in which two of the five tested *Porphyromonas gingivalis* (*P. gingivalis*) strains acquired resistance to CHX, resulting in 2–4 folds of increase in the MIC of CHX^43^. Furthermore, *Enterococcus faecalis* (*E. faecalis*) was also shown to develop drug resistance to CHX with ten repeated exposures^29^. In addition, a study reported that cariogenic bacterium *S. mutans* developed resistance to fluoride *in vitro*^44^. Therefore, bacterial resistance against widely used agents such as CHX and fluoride in the oral environment does appear to be a common problem. Other oral bacteria have also been shown to develop drug resistance. For example, *E. faecalis*, which is associated with post-endodontic treatment failures, has shown resistance to several antibiotics, including vancomycin (inhibit cell wall synthesis of bacteria) and rifampin (inhibit bacterial RNA synthesis)^45^. Therefore, the development of antibiotic resistance in the oral flora is indeed a major concern.

Bacterial resistance can be considered of being either intrinsic or acquired. Intrinsic resistance is a natural property of an organism resulting in decreased susceptibility to an antibiotic. Acquired resistance occurs due to the mutation of normal cellular genes, the acquisition of foreign resistance genes, or a combination of the two^28^. Most drug resistance mechanisms are associated with hyperexpression or acquisition of efflux pumps that actively remove the antibiotic from the membrane, or linked to the reduced permeability or stabilization of the membrane through modifications in phospholipids or membrane proteins^28,46^. However, few studies exist on the relationship between the oral bacterial drug resistance mechanisms and QAMs. One study indicated that *S. aureus* experienced a two- to four-fold increase in MIC against QAMs, with aromatic moieties suggesting that the QAM resistance mechanism was possibly related to the aromatic moieties^46^. A literature search revealed no other report on oral bacterial drug resistance against QAMs. There were several studies on bacterial drug resistance in species including *Pseudomonas aeruginosa*, *Escherichia coli*, and *Staphylococcus aureus*^47–49^. Though these are not oral bacteria, the reported drug resistance mechanisms involving efflux pumps and bacterial genetic physiological changes may also be applicable to oral bacteria. Further studies are needed to elucidate the mechanisms of bacterial resistance to QAMs, for example, on possible mutant genes and metabolic pathways that may induce changes in oral bacteria.

It is interesting that DMADDM induced bacterial drug resistance in *S. gordonii* while DMAHDM did not. Both DMADDM and DMAHDM are membrane-active antibacterial agents that interact with the cytoplasmic membranes. The antibacterial mode of action of quaternary ammonium is related to the alkyl chain length (CL). Longer chains can more readily penetrate bacterial cells, like a needle bursting a balloon. Previous studies showed that DMAHDM (CL 16) had a greater antibacterial efficacy than DMADDM (CL 12)^50^. *S. gordonii* developed drug resistance to DMADDM, but not to DMAHDM, which suggests that the drug-resistant ability in bacteria differs with different QAMs. It is possible that the bacterial membrane of the resistant strains could congeal or thicken to resist the destruction of biocide alkyl action and prevent membrane leakage. Indeed, resistant mutant strains emerged with possible changes in their susceptibility to DMADDM. However, DMAHDM could still cause bacteria lysis by penetrating the hydrophobic bacterial membrane with its longer alkyl chain length, indicating that it may be harder for bacteria to acquire resistance against longer chains. Therefore, longer alkyl chains are beneficial not only because of greater antimicrobial potency, but also because they may render it more difficult for the bacteria to develop drug resistance. The possible important relationship between the alkyl chain length and the degree of difficulty for bacteria to acquire drug resistance requires further study.

The human oral biofilm is a complex microbial community, consisting of more than 700 different bacterial species, among which approximately 20% are oral streptococci^51^. *Streptococcus salivarius*, which colonizes the oral cavities of newborns and exists in the adult oral early microbial community, develops resistance to antibiotics such as erythromycin and tetracycline^52,53^. The present study investigated three caries-related oral species: *S. gordonii, S. sanguinis*, and cariogenic *S. mutans*, which were exposed to DMAHDM resin for 20 passages, with the results that 3% DMAHDM resin could effectively inhibit most of the bacteria in the biofilm without resistant bacteria accumulation. However, the results of this study were obtained using a mass fraction of 3% DMAHDM; further study is needed to confirm if the conclusion of DMAHDM causing no drug resistance in bacteria still holds true when using different mass fractions of DMAHDM. The present study has other limitations. First, the experiments were carried out with triplicates at the same time, following previous studies^43,54^. Second, besides oral Streptococcal species, Gram negative strains and other Gram positive strains were not tested. Further studies are needed to repeat the experiments on different days to verify the drug resistance results, as well as to investigate Gram negative strains and other Gram positive strains that are important in dentistry.

Compared to planktonic bacteria, biofilms are highly tolerant of antibacterial agents and the bacteria inside the biofilm may exhibit slower growth rate and slower gene expression^55,56^. The biofilm matrix, mainly consisting of extracellular polymeric substances (EPS), contributes to the overall architecture and the resistance phenotype of biofilms. The three bacterial species used in the present study could form biofilms readily and establish mature biofilm community after culturing for 2 days, with viable CFU counts reaching 10^9^ to 10^10^ CFU/mL, as shown in previous studies^56^. It was reported that a resin containing 3% DMAHDM suppressed dental plaque biofilm formation on its surface, and reduced the EPS production by biofilms^57^. In addition, the antibacterial resin inhibited biofilms not only on its surface, but also in the outer, more remote parts of the biofilm; this was attributed to the bacterial lysis on resin surface possibly functioning as a stressful condition that triggered the programmed cell death in bacteria further away in the biofilm^58,59^. Furthermore, it has been suggested that persisters, a transient and non-hereditary phenotype without genetic changes, could be responsible for the tolerance of cells in biofilms to antibiotics^60^. A recent study showed that *S. mutans* persisters were induced after exposure to DMAHDM; however, higher doses of DMAHDM were capable of eliminating the *S. mutans* persisters^61^. The resin disks in the present study were immersed in water with stirring for 24 h to remove any unpolymerized monomers to avoid their interference with the subsequent antibacterial testing. However, it is possible that further monomer release from resin during the 48 h culture may have slightly affected the biofilms. Further study is needed to compare the biofilms grown on resin disks pre-soaked for different time periods, for example, ranging from no pre-soaking to 1 d, 3 d, and 7 d of pre-soaking, to determine the possible effects of monomer release on biofilm properties.

Besides eliminating the entire biofilm, another important approach to suppressing dental caries is to modulate, not kill, the biofilm from a cariogenic and acidogenic composition toward a benign and healthy biofilm. Indeed, from an oral microecological perspective, it is more feasible and more beneficial to maintain a healthy ecological stability for the biofilm than to eliminate the entire biofilm^62^. A recent study demonstrated that an adhesive resin containing DMADDM could change the species composition in an oral streptococci biofilm, by reducing the pathogenic species percentage and increasing the healthy species percentage^39^. Preliminary experiments in our laboratory showed that incorporating DMAHDM into a composite modulated the biofilm toward a healthy composition; the cariogenic *S. mutans* proportion in the biofilm on DMAHDM composite was much lower than that on control composite, where an overwhelming proportion of *S. mutans* existed in the biofilms. Therefore, dental resins containing DMAHDM may have triple benefits: (1) Antibacterial activity; (2) not inducing drug resistance in bacteria; and (3) modulating biofilm species toward a healthy composition. Potential applications for resins containing DMAHDM may include adhesives, composites, cements, coatings, and pit and fissure sealants, to possess antibacterial and biofilm-modulating capabilities with no drug resistance. Further *in vitro* and *in situ* evaluations in human participants are needed to investigation these applications.

## Conclusion

This study investigated the antibacterial drug resistance of *S. mutans*, *S. sanguinis* and *S. gordonii* against quaternary ammonium compounds, and tested DMAHDM in a dental resin with biofilms having repeated exposures for 20 passages. The survived bacteria from the previous passage were used as inoculum for the subsequent passage. *S. gordonii* acquired drug resistance to CHX. However, neither *S. mutans*, nor *S. sanguinis*, nor *S. gordonii* developed any drug resistance to the new antimicrobial monomer DMAHDM with repeated exposures for 20 passages, in both planktonic and biofilm forms. The antibacterial potency of the resin containing 3% DMAHDM remained high against the three streptococcal bacterial biofilms, achieving biofilm CFU reductions of 3–4 log, with no change from 1 to 20 passages. Hence, the new DMAHDM with potent antibacterial activities and no drug resistance is promising for dental applications to control oral biofilms and inhibit caries.
